# Observation of a relativistic analog of the Brewster effect in electron-photon scattering

**DOI:** 10.1038/s41467-026-76013-5

**Published:** 2026-07-28

**Authors:** Brian Schaap, Maximilian Lenz, Pietro Musumeci

**Affiliations:** Department of Physics and Astronomy, University of California at Los Angeles, Los Angeles, CA USA

**Keywords:** Optical physics, Experimental particle physics

## Abstract

The scattering of light by relativistic charged particles is a fundamental process in electrodynamics, underpinning phenomena ranging from cosmic ray interactions to laboratory X-ray generation. While the spectral and temporal properties of this process are well understood, the polarization-dependent response as a function of incidence angle has remained largely unexplored. Here we report the experimental observation of a relativistic analog to the Brewster effect in the interaction between a high-brightness relativistic electron beam and an optical laser pulse at shallow incidence. For incident radiation polarized in the plane of scattering, Lorentz-transformations predict a distinct interaction angle at which photon emission is suppressed in the forward direction, in close analogy with Brewster reflection at dielectric interfaces. These results connect a central concept of classical optics to the relativistic electron-photon scattering and highlight polarization as a controllable degree of freedom in light-matter interactions involving high-energy particle beams.

## Abstract (web summary)

Polarization dependent phenomena like the Brewster effect have remained unexplored in the relativistic regime. Here, the authors report the observation of a relativistic analog of the Brewster effect within relativistic electrons colliding with an optical pulse at shallow incidence.

## Introduction

Polarization by reflection is a ubiquitous optical phenomenon, encountered not only in laboratories but in everyday experience, from the suppression of ocean glare to intraspecific signaling of butterflies^1^. This familiar effect is formalized by Brewster’s law, which states that for light incident on an interface between two media, there exists a characteristic incidence angle at which the reflected field becomes fully polarized perpendicular to the plane of incidence^2^. For isotropic dielectric media with refractive indices *n*_1_ and *n*_2_, the Brewster angle is given by $\tan {\theta }_{{{{\rm{B}}}}}={n}_{2}/{n}_{1}$^3^. Under these conditions, the in-plane (*p*-polarized) reflection channel vanishes, producing complete suppression of one polarization component.

Generalizations to relativistic dielectric^4,5^, plasma^6^ continua, temporal metamaterials^7^, and engineered metasurfaces^8–11^, including recent demonstrations of anomalous Brewster absorption^12^ and full-polarization omnidirectional Brewster effect^13^, show that polarization-controlled suppression of reflection persists well beyond the limits of classical ray optics. In these macroscopic systems, the Brewster condition is achieved by tailoring the medium's electric and magnetic susceptibilities to achieve polarization-dependent impedance matching. Viewed microscopically, the effect arises from the radiation pattern of the induced electric and magnetic dipoles: when the direction of charge oscillation aligns with the direction of observation, emission is forbidden, yielding a geometrically enforced zero in the scattered field. Brewster’s law, therefore, reflects a broader symmetry constraint in light-matter interactions, suggesting that analogous radiation zeros should persist in more general media and, ultimately, even in the absence of a material interface.

The most elementary realization of this idea is the scattering of light from a free relativistic electron. In this case, the interaction is governed by the simultaneous action of the electric and magnetic Lorentz forces on the moving charge. At a specific interaction geometry, the transverse components of the electric and magnetic forces induced by a *p*-polarized electromagnetic wave perfectly balance. This velocity-dependent cancellation plays a role analogous to impedance matching, but arises purely from relativistic kinematics rather than from a material constitutive response. Under these conditions, the driven charge motion aligns along the propagation axis, thereby suppressing the forward emission. Specifically, for an electron with velocity *βc* and Lorentz factor *γ*, the suppression occurs at the angle

1 $$\tan {\theta }_{{{{\rm{B}}}}}=\frac{1}{\gamma \beta },$$

for which the scattering cross-section collapses to zero along the electron beam axis. We identify this singularity as a relativistic analog to the Brewster effect.

Despite the prevalence of electron-photon interactions in modern physics, its Brewster-like polarization response in the relativistic regime has never been experimentally isolated. The scattering process where the photon gains energy from the electron, also referred to as inverse Compton scattering, is routinely utilized to generate high-brightness X- and *γ*-rays in compact setups^14–23^. However, investigations in this field have focused almost exclusively on the radiation spectral properties resulting from the large relativistic Doppler shift in the counterpropagating regime, on the optimization of photon yield, and the angular distribution of the scattered light. While polarization is known to modify the angular distribution of Compton radiation^24^, the Brewster-like on-axis node that emerges at shallow-angle scattering from p-polarized electromagnetic waves has not been explicitly observed in this relativistic inverse-Compton geometry.

Here, we report the direct observation of a relativistic analog to the Brewster effect in electron-photon scattering. By colliding a high-brightness electron beam with a linearly polarized laser pulse in a shallow-angle overtaking geometry^25–29^, we access a kinematic regime where the polarization response of the scattering process becomes the dominant observable. We demonstrate that while *s*-polarized light scatters efficiently, the on-axis emission of *p*-polarized photons is extinguished at the predicted Brewster angle. Our measurements, supported by analytical theory, confirm that the scattering from a relativistic electron beam behaves analogously to Fresnel reflection off a dielectric medium. These results provide a direct visualization of the Lorentz-transformed dipole radiation pattern and demonstrate polarization-gated response in relativistic light-matter interactions.

## Results

### Origin of the scattering node

**Fig. 1:**
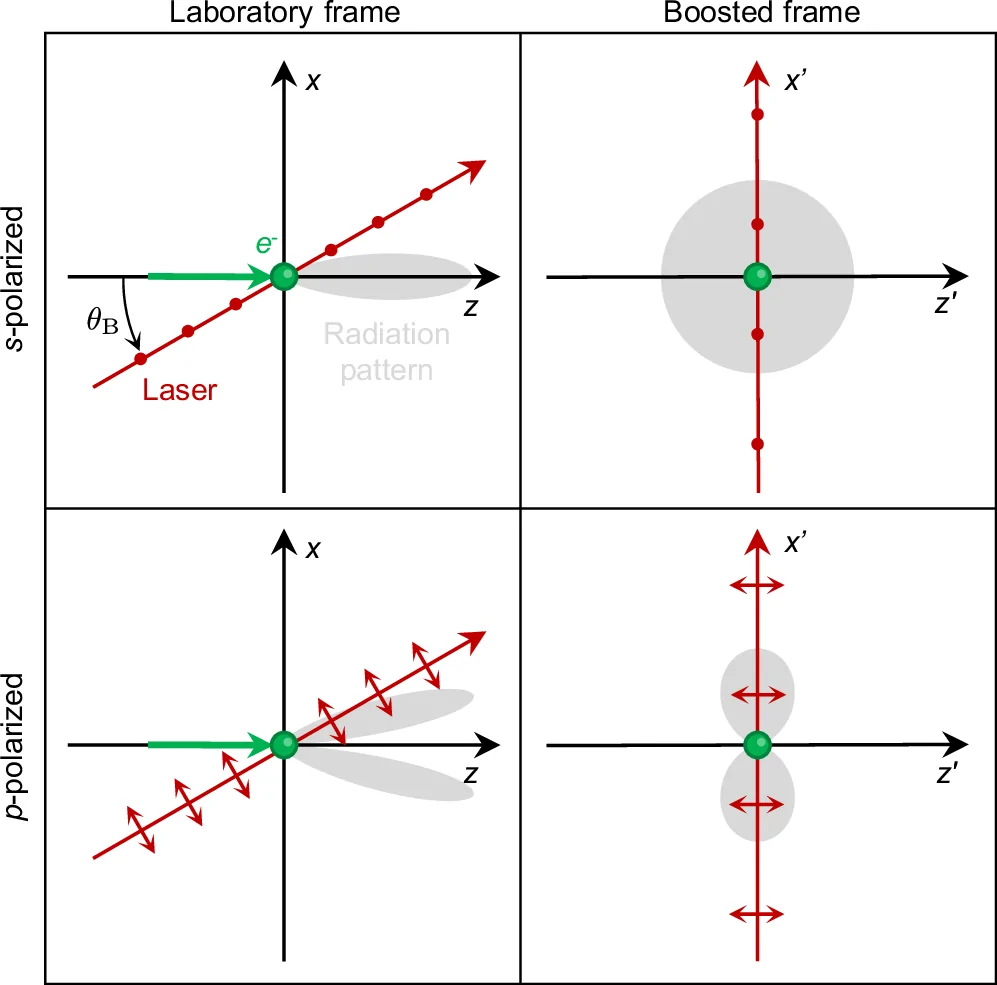
Kinematics of the relativistic Brewster Effect. *Left:* In the laboratory frame, the electron (green) and laser (red) cross at the Brewster angle *θ*_B_. *Right:* In the rest frame the Lorentz transformation maps to normal incidence. *Top:* For s-polarization, the electron oscillates transversely, allowing efficient scattering along the propagation axis. *Bottom:* For p-polarization, the electron dipole oscillates along the observation axis, suppressing forward emission.

To understand the physical mechanism behind this suppression, it is instructive to consider the interaction in the instantaneous rest frame of the electron (primed coordinates), where the particle is driven by the Lorentz-transformed laser field. In this frame, the angle of incidence transforms via relativistic aberration as $\tan \theta {\prime}=\sin {\theta }_{0}/[\gamma (\beta -\cos {\theta }_{0})]$^30^. The relativistic Brewster angle corresponds to the laboratory crossing angle that maps to normal incidence in the rest frame. For an ultrarelativistic beam, this condition is approximated by *θ*_B_ ≃ 1/*γ*. Figure 1 depicts the radiation pattern resulting from *s*- and *p*-polarization in both frames at the Brewster condition. For an *s*-polarized laser pulse, the induced dipole oscillation in the rest frame is transverse to the observation axis (concentrated in the $x{\prime} z{\prime}$-plane). In the laboratory frame, this transforms into the conventional inverse Compton radiation pattern, which is peaked along the electron beam axis. However, for *p*-polarization at *θ*_B_, the induced dipole moment aligns exactly parallel to the rest-frame $z{\prime}$-axis. Since a dipole does not radiate along its oscillation axis, there is no energy emitted in the direction of the electron beam. In the laboratory frame, this node maps to a vanishing on-axis signal and an annular radiation pattern.

**Fig. 2:**
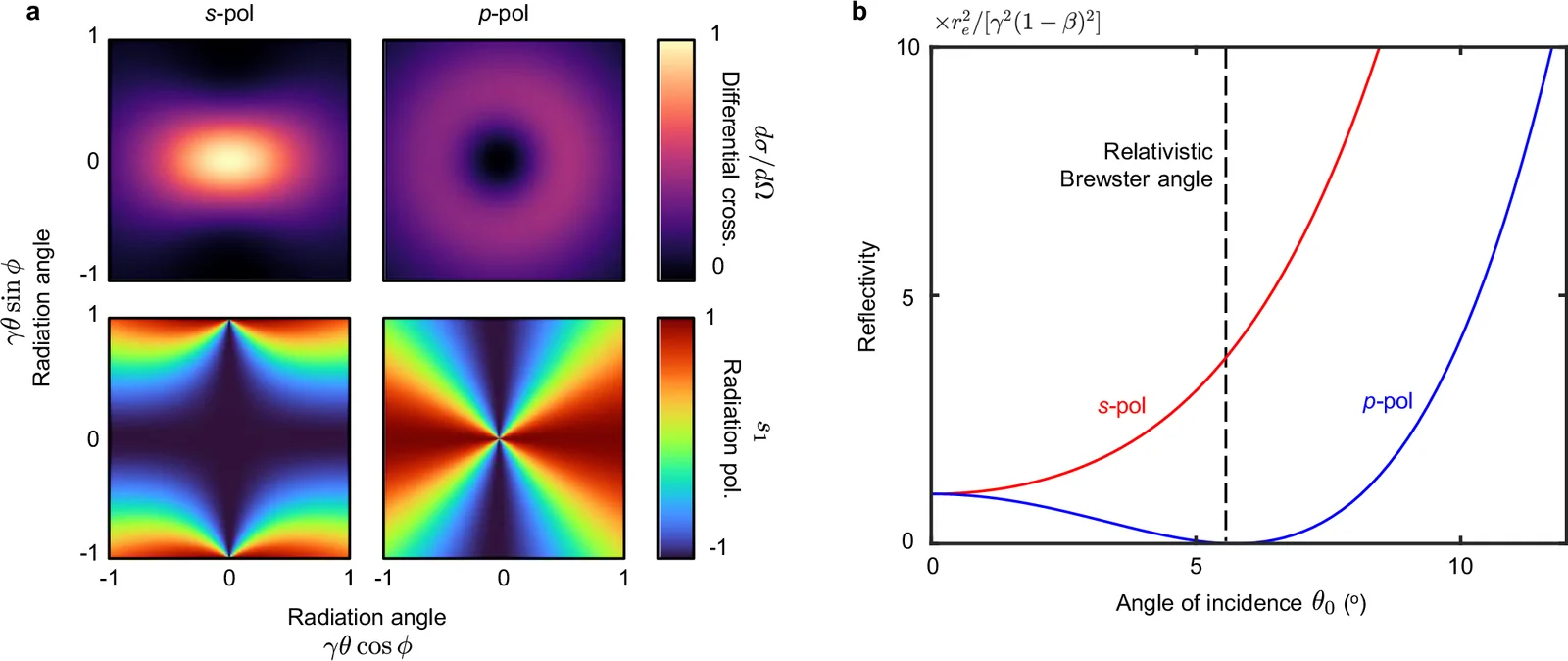
Distribution of the scattered radiation. **a** Distribution of the scattered radiation.*Top:* Differential cross-sections *dσ*/*dΩ* in the laboratory frame. While *s*-polarization (*left*) yields a standard emission cone peaked on-axis, *p*-polarization (*right*) at the Brewster angle exhibits a conical shape with a vanishing on-axis signal, corresponding to the direction of the relativistic dipole oscillation. *Bottom:* Degree of linear polarization shows the transition from linear to radial polarization, forcing a vanishing electric field along the beam axis. **b** The relativistic reflectivity curve. On-axis reflectivity $R={P}_{{{{\rm{out}}}}}/{P}_{{{{\rm{in}}}}}=({\omega }_{{{{\rm{rad}}}}}^{2}/{\omega }_{0}^{2})d{\sigma }_{\theta=0}/d\Omega$ as a function of the crossing angle, constructing the relativistic analog to the Fresnel reflection curves of a dielectric interface. While the *s*-polarized scattering (red curve) remains robust across the angular range, the *p*-polarized scattering (blue curve) vanishes at the Brewster angle. The experimental measurement at 5. 8^∘^ sits precisely within this theoretical dip.

This relativistic Brewster analog is thus not merely a reduction in scattering efficiency; it is a fundamental topological transition in the radiation distribution dictated by special relativity. Quantitatively, this behavior is described by the relativistic differential cross-section in the laboratory frame (see Methods). While for *s*-polarization it reduces to the well-known form for inverse Compton scattering $d\sigma /d\Omega={r}_{e}^{2}/{[\gamma (1-\beta \cos \theta )]}^{2}\{1-{\sin }^{2}\theta {\sin }^{2}\phi /{[\gamma (1-\beta \cos \theta )]}^{2}\}$^23,31,32^ with *ϕ* the azimuthal angle measured from the polarization axis, shown in Fig. 2a, for *p*-polarized incidence at the Brewster angle, the scattering cross-section condenses to the anomalous form:

2 $$\frac{d\sigma }{d\Omega }=\frac{{r}_{e}^{2}{\sin }^{2}\theta }{{\gamma }^{4}{(1-\beta \cos \theta )}^{4}},$$

where *r*_*e*_ is the classical electron radius and *θ* is the scattering angle measured from the propagation axis of the electron. This function represents an annular radiation pattern which is strictly zero on-axis, i.e., the scattering node, and maximizes at an off-axis scattering angle of about ${\theta }_{{{{\rm{max}}}}}=\pm 1/(\sqrt{3}\gamma )$. Interestingly, the polarization of the annular radiation changes from linear, as is usual for inverse Compton scattering from a linear polarized laser pulse^24^, to radial with a singularity on-axis. In the bottom row of Fig. 2a, this is shown by plotting the Stokes parameter *s*_1_ = (∣*E*_*x*_∣^2^ − ∣*E*_*y*_∣^2^)/(∣*E*_*x*_∣^2^ + ∣*E*_*y*_∣^2^) with *E*_(*x*, *y*)_ the electric field components of the emitted radiation in the *x* and *y*-direction, for both incident laser polarizations.

To rigorously establish the analogy with classical optics, we mapped the on-axis scattering efficiency against the interaction angle, constructing the relativistic equivalent of a Fresnel reflection plot (Fig. 2b). We calculated the reflectivity $R={P}_{{{{\rm{out}}}}}/{P}_{{{{\rm{in}}}}}=({\omega }_{{{{\rm{rad}}}}}^{2}/{\omega }_{0}^{2})d{\sigma }_{\theta=0}/d\Omega$ as a function of the collision angle *θ*_0_, where *ω*_rad_ is the relativistic Doppler shifted frequency of the reflected radiation which on-axis is given by ${\omega }_{{{{\rm{rad}}}}}={\omega }_{0}(1-\beta \cos {\theta }_{0})/(1-\beta )$. The quadratic scaling with frequency arises from two distinct relativistic effects: first, the energy per photon *ℏω*_rad_ is shifted by the Doppler factor, and second, the scattered pulse is temporally compressed by the same factor, resulting in a corresponding enhancement in peak power. The resulting curve exhibits the classic Brewster signature: while the *s*-polarized scattering remains robust across the angular range, the *p*-polarized scattering vanishes at the relativistic Brewster angle.

### Observation of the polarization response

The signature of the relativistic analog to the Brewster effect is a dramatic anisotropy in the scattering efficiency, which was experimentally probed at the UCLA Pegasus photoinjector by colliding 4.7 MeV electron bunches with 780 nm laser pulses at a shallow incidence angle of *θ*_0_ = 5. 8^∘^, corresponding to the Brewster condition. For this interaction, the Doppler shift is relatively small, and most of the radiation emission is in the visible range. To detect the ICS photons, while maximizing the signal-to-noise ratio, we used a band-pass filter transmissive only in the 400–720 nm window and demagnified the source plane on an intensified CCD detector (see Methods for more details).

**Fig. 3:**
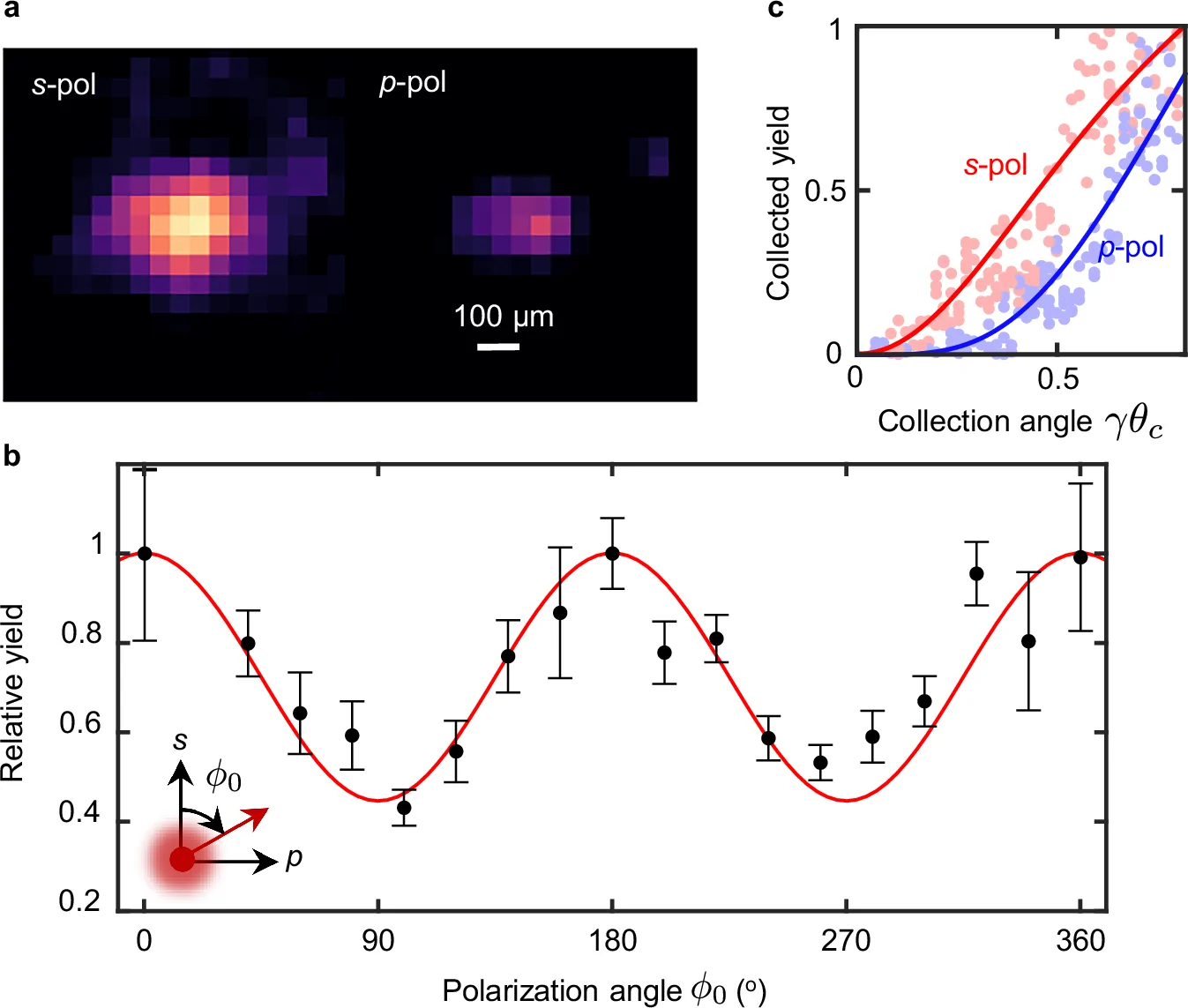
Experimental observation of the relativistic Brewster node. **a** Source imaging. Transverse intensity profiles of the scattered radiation captured at the image plane. While *s*-polarized incidence (*left*) results in a bright, spatially confined signal at the interaction point, *p*-polarized incidence (*right*) exhibits a dramatic suppression of the intensity, indicating the presence of the scattering node. **b** Polarization gating. Photon yield relative to *s*-polarization measured as a function of the laser polarization angle *ϕ*_0_. The data (black circles) follows a high-contrast ${\cos }^{2}{\phi }_{0}$ modulation (red solid curve), demonstrating that the electron beam acts as a relativistic polarizer. The error bars indicate one standard deviation in the relative number of counts. The finite residual signal for p-polarization reflects the predicted annular redistribution of the radiation field and is well predicted by integrating the analytic differential cross-section over the finite detector aperture *γθ*_*c*_ = 0.5. **c** Radiation distribution. Integrated photon yield as a function of the collection half-angle *θ*_*c*_. The *s*-polarized signal (red) rises rapidly, consistent with a forward-peaked emission cone. In contrast, the *p*-polarized signal (blue) shows a delayed onset, remaining suppressed until *γθ*_*c*_ ≃ 0.3. This distinct slope, which agrees well with the cumulative integral of the differential cross-section $\int_{0}^{{\theta }_{c}}\int_{0}^{2\pi }d\sigma /d\Omega \sin \theta d\theta d\phi$, confirms that the radiation is not simply attenuated, but redistributed into off-axis lobes, leaving a node in the center of the emission pattern.

We first visualize the change in efficiency by toggling the incident laser polarization between the *s*-state and *p*-state. For *s*-polarized incidence, the imaging system records a bright, spatially confined spot corresponding to the electron beam distribution (Fig. 3a). However, upon rotating to *p*-polarization, the signal intensity drops significantly. To systematically quantify this suppression, we performed a continuous scan of the laser polarization angle *ϕ*_0_. Figure 3b displays the measured photon yield (red stars) as a function of *ϕ*_0_. The data exhibits a high-contrast ${\cos }^{2}{\phi }_{0}$ modulation, transitioning from the maximum at *s*-polarization to the deep minimum at *p*-polarization. The finite extinction ratio is a direct consequence of the annular redistribution of the p-polarized radiation: while the node remains on axis, the detector aperture collects part of the off-axis emission (shown in Fig. 2a). Integrating the analytic differential cross-section over the experimental collection angle (*γθ*_*c*_ = 0.5 in this measurement) quantitatively reproduces the observed modulation depth. The excellent agreement between the data and the theoretical prediction confirms that the electron beam acts as a relativistic polarizer.

While the on-axis signal vanishes for *p*-polarization, the scattering cross-section is not zero everywhere; rather, the radiation is redistributed in an annular pattern described by Eq. (2). To map this topological change of the radiation distribution, we analyzed the accumulated photon counts as a function of the detector collection half-angle *θ*_*c*_ (Fig. 3c). The yield for *s*-polarized light rises rapidly, consistent with the characteristic 1/*γ* emission cone of relativistic scattering. In contrast, the *p*-polarized yield remains suppressed until a collection angle of approximately *γθ*_*c*_ = 0.3. The distinct slope of the *p*-polarized curve, in good agreement with the cumulative integral of Eq. (2), indicates that the emission has been pushed into off-axis lobes, leaving a node in the center of the radiation pattern. This measurement confirms the relativistic Brewster dip as a topological feature of the radiation field.

## Discussion

While the relativistic analog of the Brewster effect limits the Compton edge to frequency doubling of the drive field, the polarization dynamics at larger shallow crossing angles remain critical for optimizing high-brightness X-ray sources. To access short wavelengths in the shallow angle geometry, the interaction requires scaling to higher electron beam energies than conventional head-on collisions, which naturally collimates the radiation cone due to relativistic beaming, yielding higher brightness^25,33^. In this regime, even far from the Brewster node, the polarization-dependent coupling breaks the cylindrical symmetry of the emission. Specifically, the radiation pattern develops an angular shift, with an off-axis maximum at ${\theta }_{{{{\rm{max}}}}}\simeq 2/(3{\gamma }^{2}{\theta }_{0})\sin {\phi }_{p}$ along the polarization direction. This deviation can be strategically exploited to spatially separate the radiation from the electron path, for e.g., non-destructive beam diagnostics, or to optimize superradiant sources by aligning the emission with the characteristic superradiant walk-off angle^34^. Furthermore, these polarization dynamics may have scale-invariant implications in astrophysical contexts, potentially influencing the polarization signatures of relativistic jets in active galactic nuclei or pulsar winds, where similar shallow-angle scattering geometries could naturally occur.

The observed topological change in radiation distribution reveals a direct pathway for the generation of structured radiation across the electromagnetic spectrum. The radially polarized beam generated at the Brewster node is mathematically equivalent to a coherent superposition of orthogonal spin and orbital angular momentum states, lying on the higher-order Poincaré sphere as $\left\vert \psi \right\rangle=(\left\vert R\right\rangle \left\vert \ell \right\rangle+\left\vert L\right\rangle \left\vert -\ell \right\rangle )/\sqrt{2}$^35^. The scattering interaction at the Brewster angle, therefore, also functions as a vortex mode converter, analogous to a spiral phase plate but driven purely by kinematic selection. While the generation of orbital angular momentum in electron-photon scattering has been thought to be restricted to the nonlinear regime^36,37^ or by orbital angular momentum transfer from a vortex laser^38,39^, our results demonstrate that structuring is accessible even in the linear regime without initial vorticity.

The response at the Brewster angle becomes more nuanced in the following two regimes. First, at high drive field intensities, specifically when the normalized laser vector potential approaches unity (*a*_0_ = *eE*_0_/*mωc* ~ 1), the transverse momentum of the electron becomes relativistic, leading to second-order coupling with the magnetic field via the **v** × **B** Lorentz force term. The resulting nonlinear response generates a spectrum of harmonics^15^, each possessing a distinct radiation topology that could allow for non-vanishing emission along the beam axis. Secondly, scattering of photons with very high photon energy, satisfying $\hslash \omega {\prime} \sim m{c}^{2}$ in the rest frame, introduces quantum recoil that can significantly perturb the momentum of the electron^40^. This potentially leads to changes in the cancellation of the *p*-polarized amplitude and introduces energy-dependent corrections. Consequently, modifications to the Brewster-like effect are expected in the nonlinear and quantum regime.

Under certain conditions, the spatial structure of the electron beam can induce superradiant emission. Typically, this requires the electron bunch length to be on the order of the emitted radiation wavelength^34,41^. However, the relativistic Brewster angle presents an interesting geometric condition: Since the laser drives the electrons from normal incidence in the electron rest frame ($\theta {\prime}=\pi /2$), the coherence of the emission depends strongly on the transverse waist of the electron beam rather than its longitudinal length. Consequently, similar to generalized superradiance from superluminal charge density modulations^42,43^, this geometry permits superradiant emission even with longer electron bunches, provided the transverse dimension in the interaction plane *σ*_*x*_ is smaller than the radiation wavelength in the rest frame *γλ*_0_. This condition is implicitly described in the general bunching formalism described in ref. ^34^. Crucially, in this coherent regime, the macroscopic shape of the electron bunch acts as the analog to a macroscopic dielectric boundary. By actively tailoring the beam geometry, such as crabbing the angle of the electron beam^44^, one can enforce specific phase-matching conditions that destructively interfere off-axis radiation, thereby dynamically changing the depth of the scattering singularity. A detailed study of this specific transverse superradiance phenomenon will be the subject of future work.

In summary, we have experimentally isolated a relativistic analog to the Brewster effect, establishing it as a fundamental mechanism for polarization control in electron-photon scattering. By mapping the radiation field at this kinematic singularity, we demonstrated that the scattering response of free electrons is geometrically structured by their momentum vector, effectively functioning as a relativistic polarizer. Beyond confirming the single-particle cross-section, this discovery offers a pathway toward the synthesis of exotic vector beams and structured light fields across the electromagnetic spectrum. More broadly, the result illustrates that the dielectric Brewster effect can be viewed as a macroscopic, collectively enhanced manifestation of a more elementary radiation-selection rule: a driven charge or induced dipole does not radiate along its oscillation direction. In the present relativistic single-particle limit, this selection rule appears as an on-axis polarization node with finite off-axis radiation, providing a route to polarization-gated and structured radiation at the source.

## Methods

### Experimental setup

**Fig. 4:**
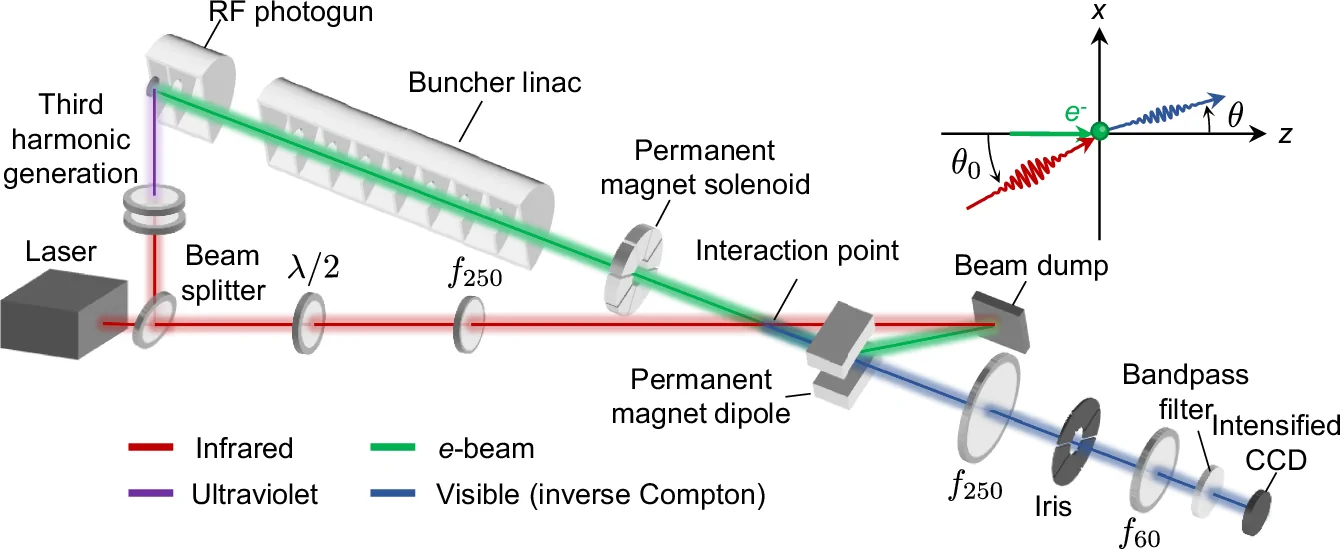
Schematic of the beamlines used in the experiment. See text for details. Certain beamline elements are adapted from the schematic in our previous work in ref. ^51^.

The experiment was carried out at the UCLA Pegasus photoinjector^45^, with the setup shown schematically in Fig. 4. A 1.6-cell S-band RF gun produces 200 pC electron bunches from a NaKSb photocathode^46^ illuminated by 100 fs UV pulses. A buncher linac then accelerates the beam to 4.7 MeV and compresses it to 300 fs rms at the interaction point. Here, the *e*-beam is focused transversely to a 150 μm waist by a radially magnetized permanent magnet solenoid^47^. The Compton laser is a 780 nm, 100 fs FWHM, 6 mJ infrared pulse, focused to a 100 μm waist at the interaction point, corresponding to a peak intensity of 3.5 × 10^14^ W/cm^2^. The interaction geometry was fixed at a shallow incidence angle of *θ*_0_ = 5. 8^∘^, chosen to satisfy the relativistic Brewster condition, which maps to a fundamental singularity in the scattering cross-section. To control the linear polarization angle *ϕ*_0_ a half-waveplate was used. After the interaction, a permanent magnet dipole deflects the *e*-beam into a dump. The emitted visible radiation is collected by a two-lens point-imaging system and directed onto an intensified CCD detector, which was calibrated using optical transition radiation from a metal foil (see Supplementary Fig. 2). The source was demagnified by 24% to increase sensitivity to low photon counts. A motorized iris in the back-focal plane of the first lens allows for angularly resolved measurements, and to improve the signal-to-noise ratio, we used a band-pass filter transmissive only in the 400–720 nm window, corresponding to the expected relativistic Doppler shift. For the spatial overlap, a removable lanex screen was used, allowing for transverse profiling of both the laser and *e*-beam at the interaction point. Temporal synchronization is obtained by adjusting a linear delay stage while monitoring the relative time-of-arrival of the bunch with respect to the laser pulse by electro-optic sampling of the *e*-beam Coulomb field in a birefringent ZnTe crystal (see Supplementary Information).

### Analytical scattering cross-section

The relativistic differential cross-section can be found by Lorentz transforming the Klein-Nishina cross-section^48^, as given in ref. ^49^. However, since in the experiment the electron beam energy is much smaller than the emitted photon energy, we can also describe the interaction between the electron and a laser pulse in the framework of classical electrodynamics. In this framework, the radiated electric field is given by the Lienard-Wiechert fields, which in the spectral domain at an observation distance *r* far away from the interaction point is given by ref. ^50^

3 $${{{{\bf{E}}}}}_{{{{\rm{rad}}}}}({{{\bf{r}}}},\omega )=\frac{i\omega {e}^{i\omega r/c}}{4\pi {\epsilon }_{0}cr}{{{\bf{n}}}}\times {{{\bf{n}}}}\times \int{{{\bf{J}}}}({{{\bf{x}}}},t){e}^{i\omega (t-{{{\bf{n}}}}\cdot {{{\bf{x}}}}/c)}dt{{{\bf{dx}}}},$$

with *ω* the angular frequency of the radiation, *ϵ*_0_ the vacuum permittivity and **n** the propagation direction of the emitted radiation. The electric current of general electron beam consisting of *N* electrons is given by ${{{\bf{J}}}}=-\mathop{\sum }_{j=1}^{N}e{{{{\bf{v}}}}}_{j}{\delta }^{(3)}({{{\bf{x}}}}-{{{{\bf{x}}}}}_{j})$ with *δ*^(3)^(*x*) the three dimensional Dirac delta function, **x**_*j*_ the electron trajectory and **v**_*j*_ the velocity. We calculate the electron trajectories for linear polarization with arbitrary polarization angle and crossing angle, with details given in the Supplementary Information. The degree of linear polarization can be directly inferred from Eq. (3). The double differential cross-section is calculated from the electric field as follows, given by *d*^2^*σ*/(*dΩdω*) = *ϵ*_0_*cr*^2^*ω*_0_∣**E**(*ω*)∣^2^/(*ωU*_0_) with *U*_0_ the laser pulse energy. By integrating over angular frequency we find the differential cross-section

4 $$\frac{d\sigma }{d\Omega }=	 \frac{{r}_{e}^{2}}{{\gamma }^{2}{(1-\beta \cos \theta )}^{2}}\left\{1-\left[\frac{\sin \theta \sin \phi \cos {\phi }_{0}}{\gamma (1-\beta \cos \theta )}\right.\right.\\ 	 \left.{\left.+\frac{(\cos {\theta }_{0}-\beta )\cos \phi \sin \theta \sin {\phi }_{0}+(\beta -\cos \theta )\sin {\theta }_{0}\sin {\phi }_{0}}{\gamma (1-\beta \cos \theta )(1-\beta \cos {\theta }_{0})}\right]}^{2}\right\},$$

which condenses to the typical form for most cases, and to the anomalous form for *p*-polarization at the Brewster angle.

The expression above describes the ideal single-particle cross-section. In the incoherent scattering regime of the present experiment, the measured signal is an incoherent sum of single-electron radiation intensities. The finite phase-space volume of the beam enters through the electron angular divergence and energy spread, which average over slightly different Brewster conditions and can therefore smear the scattering node. Electron-electron interactions affect this response only indirectly, through their upstream influence on the beam emittance and energy spread, rather than through a collective optical susceptibility during the scattering event. To preserve a high-contrast node relative to the off-axis lobes, the relative energy spread and angular divergence must satisfy *σ*_*γ*_/*γ* ≪ 1 and *γσ*_*θ*_ ≪ 1, respectively. Both of these conditions are satisfied by the beam parameters in our experiment. This convolution of the single-particle cross-section with the beam phase space, alongside the finite collection aperture of the detector, fully accounts for the finite extinction ratio observed in the measurement.

## Supplementary information


Supplementary Information
Transparent Peer Review file


## Data Availability

The data generated in this study have been deposited in the Figshare database under accession code 10.6084/m9.figshare.32774781 (https://doi.org/10.6084/m9.figshare.32774781).

## Code availability

The scripts used for the data analysis and to generate the theoretical plots are available from the corresponding author on request.
