# Supplementary material for: Observation of a relativistic analog of the Brewster effect in electron-photon scattering
Source: Nat Commun. 2026 Jul 28;17:9158. doi: 10.1038/s41467-026-76013-5 (PMC13521895; doi:10.1038/s41467-026-76013-5)
Supplement: Supplementary file 1 [file 41467_2026_76013_MOESM1_ESM.pdf]

Supplementary information to observation of a relativistic analogue of the Brewster effect in electron–photon scattering

B.H. Schaap,[1] M. Lenz,[1] and P. Musumeci[1]

[1] *Department of Physics and Astronomy, University of California at Los Angeles, Los Angeles, California 90095, USA*

(Dated: July 13, 2026)

In this Supplementary Information to "Observation of a relativistic analogue of the Brewster effect in electron–photon scattering" we first present auxiliary experimental methods including the calibration of the photon yield using transition radiation and the electric optic sampling diagnostics employed for synchronization. Then, the analytical calculations of Compton scattering from a linear polarized laser pulse with arbitrary polarization angle and crossing angle are presented.

EXPERIMENTAL CALIBRATION

Detection calibration

Spectral detection sensitivity

The measured photon yield depends on the spectral detection sensitivity (SS) of the Compton diagnostic line. The SS is given by the product of the quantum efficiency (QE) of the camera and the transmission efficiency (TE) of the optics. The QE per wavelength of the PIMAX-4 iCCD detector is indicated by the black curve in Supplementary Fig. 1, which peaks around 500 nm wavelength at about 50%. The TE is largely determined by the visible band pass filter which is obtained using a combination of commercial filters. The aggregate TE of the bandpass filter has significant TE for wavelengths between 400 and 720 nm as shown by the red curve in Supplementary Fig. 1.

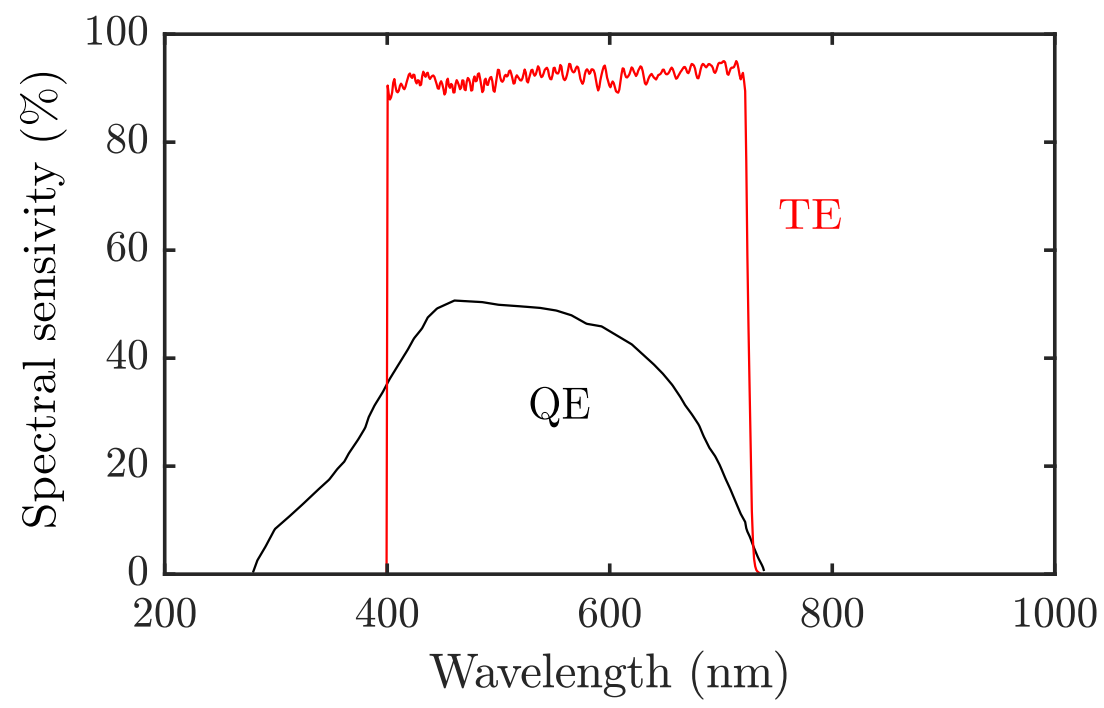

Supplementary Fig. 1. (Black) Quantum efficiency of the detector and (red) transmission efficiency of the band pass filter per wavelength.

Transition radiation

The sensitivity of the diagnostic line was calibrated using optical transition radiation (TR) from a metal foil placed in the IP. Transition radiation is the rearrangement of the Coulomb field of the electrons due to a sudden change in boundary conditions when it moves from one medium to another. Using TR as a calibration source is particularly convenient as angular and spectral characteristics are well known and similar to the Compton radiation under study. In this case, we collect the frontward radiation from the electron beam exiting the backside of a metal foil with the normal of the foil surface lying along the axis of the beam as shown schematically in the top inset of Supplementary Fig. 2.

The spectral angular photon distribution of transition radiation from an electron crossing the metal-vacuum interface can be written as [1]:

$$\frac{d^2 N_{\mathrm{ph}}^{\mathrm{TR}}}{d\omega d\Omega} = \frac{\mu_0 c e^2}{4\pi^3 \hbar\omega} \frac{\beta^2 \sin^2\theta}{(1-\beta^2\cos^2\theta)^2}, \tag{1}$$

The collected TR yield per electron is given by:

$$N_{\mathrm{ph}}^{\mathrm{TR}} = 2\pi \int_0^{\theta_c} \sin\theta d\theta \int_0^{\infty} \mathrm{SS}(\omega) \frac{d^2 N_{\mathrm{ph}}^{\mathrm{TR}}}{d\omega d\Omega} d\omega, \tag{2}$$

The optical diagnostic line downstream of the interaction is a two lens point imaging system with a demagnification of 24% to maximize signal to noise ratio on the pixels of the ICCD Princeton Instruments camera. The first lens after the interaction is a 2" diameter 30 cm focal length lens which defines the maximum collection angle to 0.09 rads. The use of shorter focal length lens to maximize the collection angle was not possible due to the space to accommodate a large gap permanent magnet dipole and eliminate the radiation noise from the electron beam hitting the optics.

For a collection angle of $\theta_c = 0.09$ rad, limited by the pupil of the second lens in the Compton diagnostic line, the SS given in the previous section and a 6.4 MeV electron beam used for the calibration the detected number of photons

per electron is $N_{\rm ph}^{\rm TR} = 1.5 \times 10^{-4}$. At the 1000 gain (linear) in the high sensitivity intensified Princeton Instruments camera used in the experiment, this resulted in about 1 ph/count (see Supplementary Fig. 2).

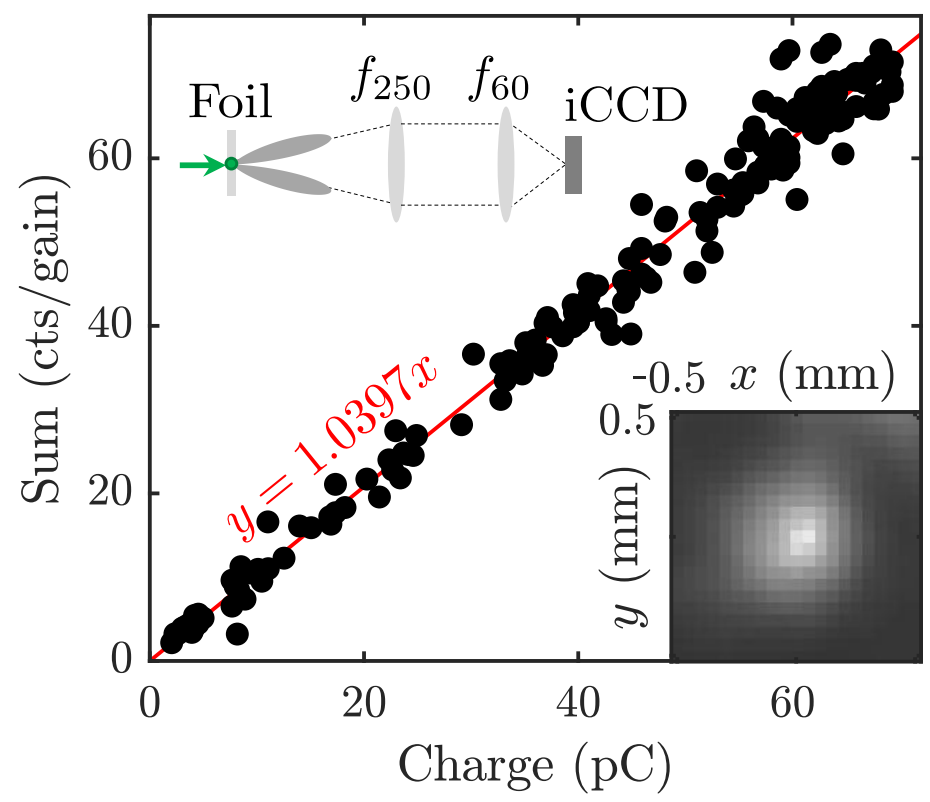

Supplementary Fig. 2. Transition radiation counts (normalized to detector gain) against bunch charge on iCCD. The red line is a linear fit though the data. Top inset: schematic of the experiment. Bottom inset: example of observed TR intensity pattern

Electric-optic sampling

As the temporal window for Compton scattering is very small, we use electro-optic sampling (EOS) of the e-beam Coulomb field as an intermediate diagnostics to assist in alignment. EOS is a commonly used technique to measure the field-induced birefringence in an electro-optic crystal using a probe infrared laser pulse, allowing direct measurements of the temporal evolution of electric fields with up to 100 fs resolution. It can be used to diagnose THz radiation [2] or even directly the Coulomb fields of a relativistic e-beam [3]. In our setup a 1 mm thick birefringent ZnTe crystal was placed at the interaction point with its normal parallel to the e-beam axis. The laser pulse was strongly attenuated to not damage the crystal or the downstream CCD detector. Before and after the interaction point crossed polarizers with a large extinction ratio are placed to maximize the sensitivity to the electric-field induced birefringence. When the e-beam and the laser pulse arrive at the same time the induced birefringence creates a polarization angle retardation on the latter given by [4]

$$\Gamma = \frac{2\pi L n_0^3 r_{41}}{\lambda_0} T_{\rm ZnTe} E_{\rm C} \tag{3}$$

in which $L = 1$ mm is the crystal length, $n_0 = 2.85$ is the refractive index of ZnTe at λ_0=780 nm and $r_{41} = 4\times10^{-12}$ m/V is the EO coefficient of ZnTe [5]. The correction coefficient $T_{\rm ZnTe}$ includes the Fresnel losses, absorption and dephasing effects which is about $T_{\rm ZnTe} \approx 0.5$ [5]. In our case the Coulomb field strength $E_{\rm C}$ is on the order of 1 MV/m, which results in retardation of about 20 degrees. The resulting increase of out-coupled laser intensity, following Malus' law, is easily detectable on a downstream CCD detector.

ANALYTICAL COMPTON SCATTERING PREDICTIONS

We describe the interaction between an electron and a laser pulse, for generality, under arbitrary crossing angle θ_0 in the framework of covariant electrodynamics, using the metric $g^{\mu\nu} = {\rm diag}(1, -1, -1, -1)$. In this classical description we neglect electron recoil, which in our case is a valid description since in the experiment the condition $\hbar\omega_{\rm rad}/(\gamma m_e c^2) \simeq 10^{-6} \ll 1$ holds, where $\hbar$ the reduced Planck's constant, $\omega_{\rm rad}$ the angular frequency of the emitted radiation, γ the Lorentz factor of the electron beam, c the speed of light and m_e the electron mass.

Present section is organized as follows: First, scattering four-potential is calculated from the trajectories of a single electron in a linearly polarized laser pulse. Then, from the four-potential, the differential cross-section is derived. Last, again using the four-potential, expressions for the electric field and polarization are found.

Derivation of scattering four-potential

The Compton radiation properties are derived starting from the far-field four-potential, which is calculated in the spectral domain from the solution to the inhomogeneous wave equation in the Lorentz gauge [6], given by

$$A^{\mu}_{\rm rad}(\mathbf{r},\omega) = \mathrm{S}(r,\omega)\sum_{j=1}^{N_e}\int u_j^{\mu} e^{ik^{\nu}x_{j\nu}}d\tau \tag{4}$$

with $\mathrm{S}(r,\omega) = (r_e/r)\exp(i\omega r/c)$ describing the spatial component of a spherical wave at position r far from the center of interaction, ω the angular frequency, r_e the classical electron radius. The summation over N_e electrons describes the spectral four-current of the electron beam with u_j^{μ} is the four-velocity of the jth electron, x_j^{μ} the four-position and $k^{\mu} = \omega/c(1,\mathbf{e}_r)$ the four-wavevector of the radiation field.

The trajectories of an electron in the laser pulse can be calculated by integrating the Lorentz force equation $\partial_{\tau}u^{\mu} = u_{\nu}\left(\partial^{\nu}A^{\mu} - \partial^{\mu}A^{\nu}\right)$ where A^{μ} here is the vector potential of the drive laser pulse. For analytical tractability we approximate the vector potential of the laser pulse used in the experiment by a Gaussian TEM_{00} mode laser beam close to its focus, neglecting beam diffraction and the radial and Gouy phase change, which can be written as

$$A^{\mu} = a_0 f(x^{\mu})\exp\left(-ik_0^{\nu}x_{\nu}\right)\epsilon^{\mu} \tag{5}$$

where $a_0 = eE_0/(m_e c\omega_0)$ is the peak normalized vector potential with E_0 the peak electric field, $k_0^{\mu} = \omega_0/c(1,\sin\theta_0\mathbf{e}_x + \cos\theta_0\mathbf{e}_z)$ is the four-wavevector of the laser pulse, $\epsilon^{\mu} = (0,\cos\theta_0\sin\phi_0\mathbf{e}_x + \cos\phi_0\mathbf{e}_y - \sin\theta_0\sin\phi_0\mathbf{e}_z)$ the polarization four-vector with ϕ_0 the polarization angle, and $f(x^{\mu})$ is a Gaussian envelope given by

$$f(x^{\mu}) = \exp\left(-W^{\mu\nu}x_{\mu}x_{\nu}\right) \tag{6}$$

where $W^{\mu\nu} = s^{\mu}s^{\nu}/(w_s^2) + p^{\mu}p^{\nu}/(w_p^2) + k_0^{\mu}k_0^{\nu}/(2\sigma_{\varphi}^2)$ with σ_{φ} the laser pulse length (in radians), $s^{\mu} = (0,\mathbf{e}_y)$ and $p^{\mu} = (0,\cos\theta\mathbf{e}_x - \sin\theta_0\mathbf{e}_z)$ four-vectors describing the transverse directions and $w_{s,p}$ the $(1/e)$ waist sizes of the laser pulse normal (s) and parallel (p) to interaction plane, respectively. Note that, if the polarization angle is $\phi_0 = 0$, the laser pulse is s-polarized, such that $\epsilon^{\mu} = s^{\mu}$, and if the polarization angle is $\phi_0 = 90^{\circ}$, the laser pulse is p-polarized, such that $\epsilon^{\mu} = p^{\mu}$.

The Lorentz force equation is integrated using a pertubative approach where the four velocity can be written as $u^{\mu} = \sum_{j=0}^{\infty}u_j^{\mu}$ where $u_n^{\mu} \propto a_0^n$. Since the vector potential amplitude of the 6 mJ laser pulse in the experiments satisfies the condition $a_0 \leq 0.0125 \ll 1$, we can evaluate the resulting trajectory equations to first order such that:

$$u^{\mu} = u_0^{\mu} + A^{\mu} - \frac{1}{\kappa}u_0^{\nu}A_{\nu}k_0^{\mu}, \tag{7}$$

where $u_0^{\mu} = \gamma(1,\beta\mathbf{e}_z)$ the initial four-velocity normalized to the speed of light and $\kappa = k_0^{\nu}u_{0\nu}$ is the central wavenumber of the laser in the instantaneous rest frame of the electron prior to interaction. The terms scaling linearly in A^{μ} describe the coupling of the electron to the electric and magnetic field of the laser pulse respectively. In Eq. (7) it is assumed that the laser pulse envelope is slowly varying with respect to the laser wavelength such that the condition $|u_{0\nu}\partial^{\nu}f(x^{\mu})| \ll |k_0^{\nu}u_{0\nu}f(x^{\mu})|$ is satisfied. The four-position can then be found by integrating over proper time τ:

$$x^{\mu} = x_0^{\mu} + cu_0^{\mu}\tau + \frac{i}{c\kappa}\left(A^{\mu} - \frac{1}{\kappa}u_0^{\nu}A_{\nu}k_0^{\mu}\right), \tag{8}$$

where ballistic sampling of the laser field was assumed so that $A^{\mu}(x^{\mu}) \simeq A^{\mu}(cu_0^{\mu}\tau)$ and the experienced laser envelope was again considered to be slowly varying with respect to the laser wavelength. Here, x_0^{μ} is the initial four-position of the electron, which for now will be assumed to be centered such that $x_0^{\mu} = (0,\mathbf{0})$. The second term describes the ballistic motion by the initial velocity of the electron and the last accounts for the induced quiver motion due to the laser field.

We can now calculate the radiation four-potential, given in Eq. (4). Before integrating, we Taylor expand the exponent to first order in A^{μ} after substituting Eq. (8):

$$\exp\left(ik^{\nu}x_{\nu}\right) \simeq \left[1 - \frac{k_{\mu}}{c\kappa}\left(A^{\mu} - \frac{1}{\kappa}u_0^{\nu}A_{\nu}k_0^{\mu}\right)\right]\exp\left(ick^{\nu}u_{0\nu}\tau\right) \tag{9}$$

By substituting Eq. (9) into the general expression for the radiation four-potential Eq. (4), while keeping only the terms that will contribute to the positive fundamental frequency, we find for a single electron that the Compton scattering four-potential becomes:

$$A^{\mu}_{\mathrm{rad}}(\mathbf{r},\omega)=\sqrt{\frac{\pi}{2}}\frac{a_0N_{\varphi}}{c\kappa}\mathrm{S}(r,\omega)\left(\bar{\epsilon}^{\mu}-\frac{k_{\nu}\bar{\epsilon}^{\nu}}{\kappa}u_0^{\mu}\right)F(k^{\mu}) \tag{10}$$

where $\bar{\epsilon}^{\mu}=\epsilon^{\mu}-\epsilon_{\nu}u_0^{\nu}k_0^{\mu}/\kappa$ is the Lorentz and gauge invariant polarization four-vector and $F(k^{\mu})$ is the linewidth function given by

$$F(k^{\mu})=\frac{1}{\sqrt{2\pi}}\frac{c\kappa}{N_{\varphi}}\int_{-\infty}^{\infty}f(u_0^{\mu}c\tau)\exp\left[i\left(k^{\mu}-k_0^{\mu}\right)u_{0\mu}c\tau\right]d\tau=\exp\left[-\frac{N_{\varphi}^2}{2}\left(1-\frac{k_{\nu}u_0^{\nu}}{\kappa}\right)^2\right], \tag{11}$$

that peaks at the resonant frequency $\omega_{\mathrm{rad}}=\omega_0(1-\beta\cos\theta_0)/((1-\beta\cos\theta)$ and rms relative bandwidth that scales with the with the inverse of

$$N_{\varphi}=\frac{\kappa}{\sqrt{2W^{\mu\nu}u_{0\nu}u_{0\mu}}}=\frac{\sigma_{\varphi}}{\sqrt{1+\frac{2\sigma_{\varphi}^2c^2\beta^2\sin^2\theta_0}{w_p^2\omega_0^2(1-\beta\cos\theta_0)^2}}}, \tag{12}$$

which is the rms interaction length in radians. The second term in the denominator can be identified as the squared ratio between the time the electron needs to traverse length of the laser pulse given by $t_k=\sigma_{\varphi}/[\omega_0(1-\beta\cos\theta_0)]$ and the time to traverse the waist (in the p-direction) of the laser pulse given by $t_p=w_p/(2^{1/2}c\beta\sin\theta_0)$. If $t_k\gg t_p$ the interaction length is determined by the finite laser waist in the p-direction.

Differential cross-section

The double differential cross-section, i.e. the fraction of photons scattered per unit solid angle, per unit spatial frequency, can be calculated from the radiation four-potential as follows

$$\frac{d^2\sigma}{d\omega d\Omega}=\frac{2\omega r^2}{\pi^{3/2}a_0^2N_{\varphi}}|A^{\nu}_{\mathrm{rad}}A_{\mathrm{rad}\nu}|=\frac{\omega N_{\varphi}r_e^2}{\pi^{1/2}c^2\kappa^2}\left[1-\left(\frac{k^{\nu}\epsilon_{\nu}}{\kappa}-\frac{k_0^{\nu}k_{\nu}u_0^{\mu}\epsilon_{\mu}}{\kappa^2}\right)^2\right]F(k^{\mu})^2 \tag{13}$$

If the interaction is long so that the conditions $N_{\varphi}\gg 1$ holds, the resonance function is rapidly varying in frequency with respect to the other components of the double differential cross-section. In that case we can use the sifting property such that $F(\omega)^2\simeq\pi^{1/2}\omega_{\mathrm{rad}}/N_{\varphi}\delta(\omega-\omega_{\mathrm{rad}})$. The differential cross-section can then be easily found by integrating over frequency which can be written as:

$$\frac{d\sigma}{d\Omega}=\frac{r_e^2}{\gamma^2(1-\beta\cos\theta)^2}\left\{1-\left[\frac{(1-\beta\cos\theta_0)\sin\theta\sin\phi\cos\phi_0+(\cos\theta_0-\beta)\cos\phi\sin\theta\sin\phi_0+(\beta-\cos\theta)\sin\theta_0\sin\phi_0}{\gamma(1-\beta\cos\theta)(1-\beta\cos\theta_0)}\right]^2\right\}, \tag{14}$$

which is also given in the manuscript. Conventionally, the cross-section of inverse Compton scattering from relativistic electrons maximizes along the beam axis. However, for shallow angle Compton scattering this is not necessarily the case anymore, which becomes clear from the on-axis differential cross-section ($\theta=0$):

$$\frac{d\sigma_{\theta=0}}{d\Omega}=\frac{r_e^2}{\gamma^2(1-\beta)^2}\left[1-\frac{\sin^2\theta_0\sin^2\phi_0}{\gamma^2(1-\beta\cos\theta_0)^2}\right], \tag{15}$$

As shown in Supplementary Fig. 3 the on-axis cross-section is constant for most cases, maximizing the radiation on-axis. However, at a particular angle of incidence given by $\theta_0=\arccos\beta=\theta_{\beta}$, the cross-section for p-polarization $\phi_0=\pi/2$ becomes zero. We refer to angle θ_{β} as the relativistic Brewster angle since it is an effect completely analogous to the well-known Brewster reflection in optics as can be ascerted comparing the Compton reflectivity $R=P_{\mathrm{out}}/P_{\mathrm{in}}=(\omega_{\mathrm{rad}}^2/\omega_0^2)d\sigma_{\theta=0}/d\Omega$ shown in the inset of Supplementary Fig. 3 with the well known Fresnel reflection curves. Physically, this is the angle at which the dipole moment aligns with the propagation axis of the electron beam. In the instantaneous rest frame of the electron (prior to interaction) the angle of incidence in this

special case is normal to the beam axis. At the relativistic Brewster angle and p-polarization ($\phi_0 = \pi/2$) Eq. (14) simplifies to

$$\frac{d\sigma^{(p,\theta_\beta)}}{d\Omega} = \frac{r_e^2 \sin^2\theta}{\gamma^4(1-\beta\cos\theta)^4}, \tag{16}$$

which describes a lobed radiation pattern peaking at angles $\theta_{\max} = \pm\arccos\{[-1+(1+8\beta^2)^{1/2}]/(2\beta)\} \pm 3^{-1/2}\gamma^{-1}$ with zero emission along the beam axis, significantly different than the usual Compton scattering cross-section. For s-polarization ($\phi_0 = 0$) Eq. (14) simplifies to the familiar expression for inverse Compton scattering:

$$\frac{d\sigma^{(s)}}{d\Omega} = \frac{r_e^2}{\gamma^2(1-\beta\cos\theta)^2}\left[1 - \frac{\sin^2\theta\sin^2\phi}{\gamma^2(1-\beta\cos\theta)^2}\right], \tag{17}$$

which a maximum along the axis ($\theta = 0$). The total cross-section found by integrating over the solid angle for both cases is equal to the Thomson cross-section $\sigma_T = 8\pi r_e^2/3$ for both cases.

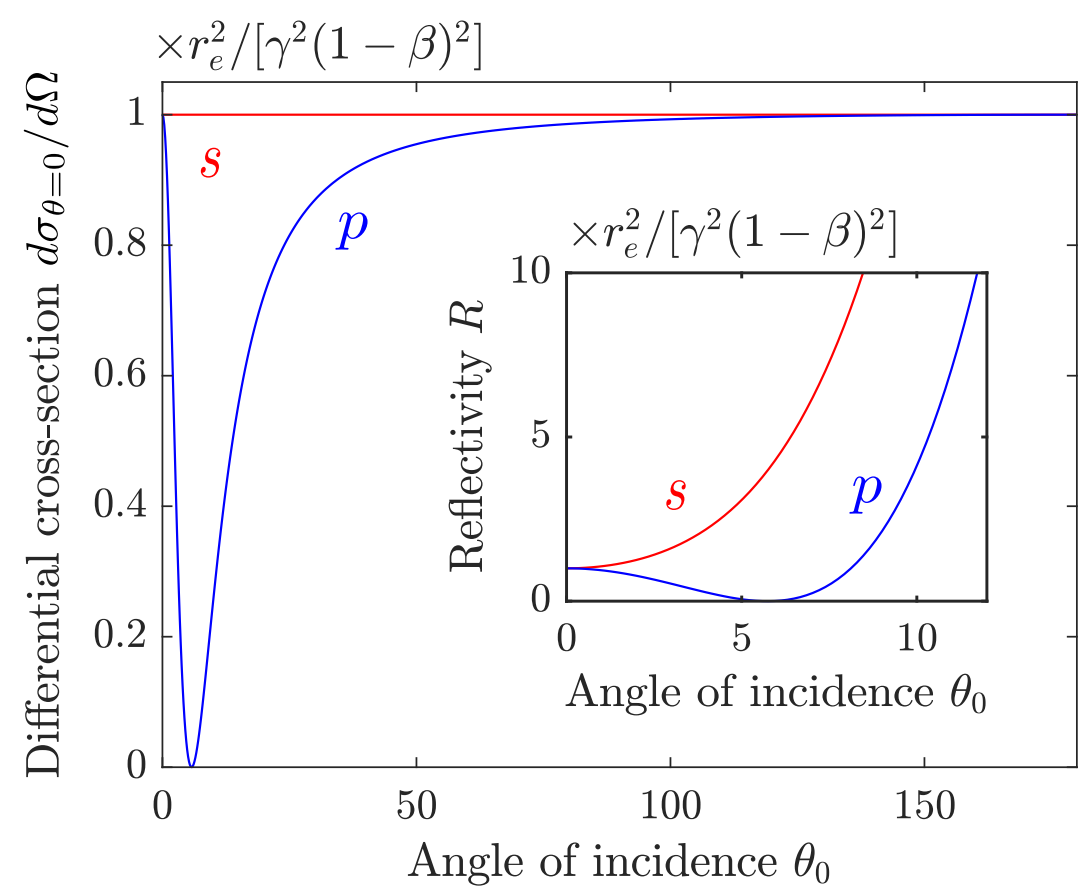

Supplementary Fig. 3. On axis differential cross-section and reflectivity (inset) versus crossing angle of the laser with an electron beam with $\gamma = 10$ ($\theta_\beta = 5.7\,\text{deg}$) for s- and p-polarization.

Photon yield

The number of photons collected within an angle θ_c can be strongly dependent on initial polarization close to the Brewster angle as discussed in the manuscript. The collected yield is proportional to the integral $N_{\text{ph}} \propto \int_0^{\theta_c}\int_0^{2\pi} d\sigma/d\Omega \sin\theta d\theta d\phi$, which for a relativistic electron $\gamma \gg 1$ at the brewster angle evaluates to:

$$N_{\text{ph}} \propto \frac{2\gamma^4\theta_c^4(3+\gamma^2\theta_c^2)}{3(1+\gamma^2\theta_c^2)^3} + \frac{(1-\gamma^2\theta_c^2)\gamma^2\theta_c^2}{(1+\gamma^2\theta_c^2)^3}\cos^2\phi_0, \tag{18}$$

where we assumed $\theta_0 = \theta_\beta$. This expression exhibits a $\cos^2\phi_0$ behavior, with a maximum for s-polarization, a minimum at p-polarization and with a modulation depth that is determined by the collection angle. In the final set of measurements we probe the polarization dependent yield given by the Eq. (18).

Degree of linear polarization

In the far field, the differential operator $d^{\mu\nu} = i\omega g^{\mu\nu} - ick^\mu u^\nu_{\text{obs}}$, where u^μ_{obs} an observer four-velocity, can be used to calculate the spectral four field $E^\mu_{\text{rad}} = d^\mu_\nu A^\nu_{\text{rad}}$. In the lab frame, where $u^\mu_{\text{obs}} = (1, \mathbf{0})$, application of this operator to the Compton scattering four-potential, given by Eq. (10), gives a temporal component equal to zero and the spatial part equal to the (normalized) spectral three field $\mathbf{E}_{\text{rad}}(r,\omega)$. Since we are only interested in the degree of linear

polarization quantified by the stokes paramemter $s_1 = (|E_x|^2 - |E_y|^2)/(|E_x|^2 + |E_y|^2)$, we will only keep track of the vectorial parts of the electric field $E_{x,y}$, since the other constant terms will factor out. The electric field vectors are given by

$$E_x = \omega\bar{\epsilon}^1 + \frac{k_\nu \bar{\epsilon}^\nu}{\kappa} u_0^0 k^1, \tag{19a}$$

$$E_y = \omega\bar{\epsilon}^2 + \frac{k_\nu \bar{\epsilon}^\nu}{\kappa} u_0^0 k^2 \tag{19b}$$

which together define the degree of linear polarization s_1.

SUPPLEMENTARY REFERENCES

[1] V. Ginzburg and I. Frank, Journal of Physics (USSR) **9**, 353 (1945).
[2] M. Lenz, A. Fisher, A. Ody, Y. Park, and P. Musumeci, Optics Express **30**, 33804 (2022).
[3] M. Ota, K. Kan, S. Komada, Y. Wang, V. C. Agulto, V. K. Mag-usara, Y. Arikawa, M. R. Asakawa, Y. Sakawa, T. Matsui, *et al.*, Nature Physics **18**, 1436 (2022).
[4] Q. Chen, M. Tani, Z. Jiang, and X.-C. Zhang, Journal of the Optical Society of America B **18**, 823 (2001).
[5] J. Van Tilborg, "Coherent terahertz radiation from laser-wakefield-accelerated electron beams," (2006).
[6] J. D. Jackson, *Classical electrodynamics* (John Wiley & Sons, 1998).
