# Supplementary material for: Observation of a relativistic analog of the Brewster effect in electron-photon scattering
Source: Nat Commun. 2026 Jul 28;17:9158. doi: 10.1038/s41467-026-76013-5 (PMC13521895; doi:10.1038/s41467-026-76013-5)
Supplement: Supplementary file 2 [file 41467_2026_76013_MOESM2_ESM.pdf]

nature portfolio

Peer Review File

Observation of a relativistic analogue of the Brewster effect in electron–photon scattering

Corresponding Author: Dr Brian Schaap

This file contains all reviewer reports in order by version, followed by all author rebuttals in order by version.

Attachments originally included by the reviewers as part of their assessment can be found at the end of this file.

Version 0:

Reviewer comments:

Reviewer #1

(Remarks to the Author)
See attachment.

Reviewer #2

(Remarks to the Author)
The authors theoretically propose and experimentally observe the phenomenon of the relativistic Brewster effect in electron-photon scattering. Fundamentally, they demonstrate a vanishing scattering of p-polarized light by relativistic electron beams. These results are novel and interesting, and the theory is preliminarily verified by experiments. Therefore, I believe this manuscript deserves publication in Nature Communications, provided the following comments and questions are adequately addressed.
1. I suggest that the structure of the electron beam be elaborated upon in greater detail, specifically regarding whether this structure plays a role in the observed effect. The beam structure might influence the orientation of electron-electron interactions within the beam (i.e., whether they are predominantly along the beam axis or perpendicular to it), which could subsequently impact its optical response.
2. The authors mention the concept of "moving media." Would it be possible to schematically plot the optical dispersion surface of this electron beam treated as a moving medium? Furthermore, what is the major consequence of the momentum shift in this effect, and how does it evolve with the speed of the electrons?
3. Impedance matching of p-polarized light at the Brewster angle is another common explanation for the Brewster effect. Following this understanding, the Brewster effect has been extensively developed in the field of metamaterials recently. Notable examples include the discovery of the full-polarization omnidirectional Brewster effect (Light Sci. Appl. 13, 262 (2024)) and the anomalous Brewster effect, which enables perfect absorption (Light Sci. Appl. 10, 89 (2021)). The readers would benefit from a comprehensive introduction to these developments and some potential discussion from the perspective of impedance matching.
4. The experimental results shown in Fig. 3 exhibit some discrepancies compared to the theoretical results in Fig. 2. What is the main cause of this deviation? Is it possible to improve the results or add more analyses to bolster confidence in the observation of this phenomenon?

Version 1:

Reviewer comments:

Reviewer #1

(Remarks to the Author)
The authors have provided answers to all my comments.

Based on their extensive reply, I feel that my initial comments have been reaffirmed in the sense that the direction of the analogy is better understood from microscopic relativistic light-matter interaction to macroscopic polarization optics and not

vice versa. As such, calling this the "relativistic Brewster effect" remains problematic in my eyes. A framing such as "analogous to the Brewster effect" would be more honest.

Similarly, the claim that "this discovery unlocks a new degree of freedom for high-energy photonics" is still overly sensational. I understand the authors try to "sell" their nice and mostly sound results to Nature Communications, but claiming that this result would now allow for people to make structured light at high photon energies using this technique is - in my view - hype. The photon numbers we are talking about here are small and the coherence regime is far away (in particular for high photon energies). The additional discussion of a path towards super-radiant emission is a neat idea, but without any simulations backing this up, it is not clear what those conditions would actually be. The yardstick for such kind of claims should be [Malaca et al. Nat. Phot. 18 (2024)], who did extensive simulations to support claims of super-radiance regimes.

I understand that the authors are looking for a high-impact venue to publish their results visibly, but I would hope that one can settle on a more honest framing that would not distract fellow researchers. In conclusion, the work appears sound and my only criticism is the framing. After an adjustment of the framing the work would be acceptable for publication in a scientific journal - whether Nat.Comms. is the right journal for this rather specific work is a question the editors have to decide on.

Reviewer #2

(Remarks to the Author)
The authors have carefully addressed my comments and revised their manuscript. I now have no more comments.

Open Access This Peer Review File is licensed under a Creative Commons Attribution 4.0 International License, which permits use, sharing, adaptation, distribution and reproduction in any medium or format, as long as you give appropriate credit to the original author(s) and the source, provide a link to the Creative Commons license, and indicate if changes were made.
In cases where reviewers are anonymous, credit should be given to 'Anonymous Referee' and the source.
The images or other third party material in this Peer Review File are included in the article's Creative Commons license, unless indicated otherwise in a credit line to the material. If material is not included in the article's Creative Commons license and your intended use is not permitted by statutory regulation or exceeds the permitted use, you will need to obtain permission directly from the copyright holder.
To view a copy of this license, visit https://creativecommons.org/licenses/by/4.0/

Point-by-Point Response to Reviewers

Reviewer 1

This manuscript is clearly written, well structured, and visually effective. The experimental presentation is careful, and the measured polarization dependence and collection-angle dependence appear to be in good agreement with the authors' model. In particular, the data do support the central experimental statement that, at the chosen shallow collision angle, the on axis signal is strongly suppressed for p-polarized incidence while remaining large for s-polarization, and that the missing on-axis intensity is redistributed into off-axis emission. That said, my main concern is not with the data quality, but with the conceptual framing and novelty claim.

Response: We sincerely thank the Reviewer for the positive assessment of our experimental data, presentation, and the pedagogical value of the Brewster analogy. We have carefully revised the manuscript to refine the conceptual framing and novelty claims as suggested.

Comment 1.1: The manuscript presents the result as the observation of the relativistic Brewster effect" and repeatedly draws an analogy to Brewster reflection at a dielectric interface, where the p-polarized reflected channel vanishes. But strictly, this is not the effect they observe: The authors show that the p-polarized scattering is not eliminated, but instead becomes an annular/lobed distribution with a zero only on axis. Both the main text and supplemental material explicitly state that the cross-section at the special angle takes the form of Eq.2 of the main text, which is zero on axis but finite off axis, peaking at a nonzero angle. First, that is not strictly analogous to the Brewster effect.

Response: We appreciate the Reviewer's distinction between the radiation pattern from microscopic scatterers and the collective response of a macroscopic dielectric interface. We agree that, in our experiment, the effect is not the complete elimination of all p-polarized scattered radiation, but rather the appearance of a polarization-dependent node along the specular/on-axis direction, accompanied by finite off-axis radiation.

The analogy to the conventional Brewster effect is based on the common microscopic origin of the node. In the dielectric case, the Brewster condition corresponds to the geometry in which the induced dipoles do not radiate along the specular reflection direction. For a macroscopic planar interface, the coherent collective response of many dipoles and the translational symmetry of the boundary restrict the reflected field to a well-defined specular plane-wave channel. As a result, the vanishing of dipole radiation in that direction appears macroscopically as the disappearance of the p-polarized reflected beam.

In our experiment, by contrast, the electron beam acts as an ensemble of independent, microscopic relativistic scatterers rather than as a continuous planar boundary. The same dipole-radiation selection rule produces a node along the corresponding on-axis direction, but the absence of a macroscopic interface to enforce destructive interference in all other directions, allows the full angular radiation pattern of the individual scatterers to be observed. This is why the p-polarized signal is not eliminated altogether, but redistributed into an annular/lobed off-axis distribution, as described by Eq. (2).

We have revised the Introduction, Results, and Discussion to make this distinction explicit. In particular, we now clarify that the term "relativistic Brewster effect" refers to the Brewster-like polarization node in the relativistic scattering pattern, not to a literal Fresnel reflection process from a relativistic dielectric interface.

Furthermore, as we now note in the revised Discussion, if this interaction could be extended

into the coherent (superradiant) regime, the macroscopic shape of the electron bunch would dictate the emergence of a collective behavior. In that limit, the electron bunch would act as a macroscopic boundary, selectively suppressing off-axis radiation through destructive interference depending on the bunch finite size. This would move the system towards the macroscopic Fresnel/Brewster limit, thereby providing an even closer relativistic analog of the conventional Brewster response.

Comment 1.2: Second, the expression above is derived from the general polarization-dependent relativistic Thomson/Compton cross-section by setting $\phi_0 = \pi/2$ and $\theta_0 = \arccos \beta$. It is thus not a new theoretical result but a particular limit of the standard formalism corresponding to the familiar p-polarized radiation pattern with an on-axis node and off-axis lobes. The radiation structure is well-documented in the literature. When the authors claim that “this fundamental optical anomaly has never been isolated”, they refer to their explicit framing. Many people have looked at the emission and seen the zero, no matter the interaction angle.

Response: We agree with the Reviewer that the general polarization-dependent relativistic Thomson/Compton cross section is well established, and we have revised the manuscript to clarify that Eq. (2) is a particular kinematic limit of a standard formalism, obtained for the shallow-angle geometry in which the p-polarized radiation pattern develops an on-axis node.

Our novelty claim is not based on the derivation of a new theoretical expression. Instead, it concerns the identification and experimental demonstration of this specific polarization-dependent kinematic node in the relativistic shallow-angle scattering regime. The referee is correct that in nonrelativistic scattering the corresponding dipole-radiation node is well known and has been well documented. Conversely, in relativistic case, while polarization-effects in Inverse Compton scattering have been observed, most relativistic inverse-Compton work has emphasized head-on or near-head-on geometries, where the goal is typically to maximize the frequency upshift and where this Brewster-like node can not be naturally isolated as an experimentally accessible observable.

We have therefore revised the text to clarify that the effect reported here is not a new term in the scattering formalism, but the first experimental observation, to our knowledge, of this particular relativistic polarization node in shallow-angle inverse Compton scattering. We have consequently softened the statement that this “fundamental optical anomaly has never been isolated” to make clear that we refer specifically to its isolation and interpretation as a Brewster-like relativistic scattering effect, rather than to the existence of polarization-dependent radiation scattering nodes in general.

Comment 1.3: What is shown here is a particularly clear and nicely visualized instance of a known polarization-dependent relativistic radiation pattern. I found it highly educational to draw the analogy to Brewster. This makes this a pedagogical achievement to me. While I highly appreciate the analogy, statements like “this work demonstrates that the foundational principles of polarization optics are not confined to material media but are intrinsic to the relativistic interaction itself” should be revised to avoid misinterpretation I am sure the authors mean it the right way, but it should be emphasized that it is not that relativistic interaction behaves like polarization optics, but that polarization optics is a special case of (relativistic) light-matter interaction. This is also why I am skeptical of the framing as ”relativistic Brewster effect”: the analogy is only partial due to the off-axis content.

Response: We thank the Reviewer for this excellent point. We have adopted the Reviewer’s phrasing directly in the conclusion. The text now emphasizes that classical polarization optics (like the dielectric Brewster effect) is a macroscopic special case of the fundamental light-matter interaction we observe here, rather than the other way around. n parallel, we have revised the

Introduction, Results, and Discussion to clarify the nature of the Brewster analogy, specifically distinguishing the observed on-axis polarization node from the finite off-axis radiation content as addressed in our response to Comment 1.1.

Comment 1.4: However, under any reading, their work does not identify a previously unknown property of electron–light scattering. Any standard Compton/Thomson simulation that retains polarization and angular dependence will already produce this node/lobe structure. This is also why I doubt that this result "establish[es] a new framework" or provides "a new degree of freedom for high-energy photonics, enabling the synthesis of exotic vector beams and structured light fields across the electromagnetic spectrum".

Response: We appreciate the Reviewer's point and fully agree that a properly polarization- and angle-resolved Compton/Thomson simulation should contain this node/lobe structure. Our point is that, although implicit in the standard formalism, this feature had remained largely unexplored because it occurs in a narrow region of parameter space. To our knowledge, it had not previously been explicitly identified, nor, most importantly, experimentally observed as a distinct relativistic scattering effect. We therefore maintain that our work makes two genuinely novel contributions:

First, while the head-on scattering regime has been extensively studied, the polarization response unique to the shallow-angle geometry was previously unrecognized in the literature. This includes both the topological transition to an annular radiation pattern exactly at the relativistic Brewster angle as well as the realization that for shallow angle interaction, increasingly common in newer ICS source designs, polarization-dependent coupling breaks the symmetry of the emission. Explicitly identifying and physically characterizing this behavior in relativistic electron-photon scattering is a fundamental milestone.

Second, recognizing this specific kinematic singularity as a mechanism to synthesize radially polarized vector beams is an entirely new concept. Generating structured light in this manner, without the need for nonlinear intensities or initial laser vorticity, could provide a novel application in high-energy photonics.

Ultimately, we concur with the Reviewer that phrases such as "establishes a new framework" overstate the theoretical scope of our work. We have therefore toned down this language in the revised manuscript. The revised text now frames our result more specifically as demonstrating the importance of polarization effects in shallow-angle inverse Compton scattering and as identifying a direct mechanism for structured-light generation, which better reflects both the fundamental and practical significance of the work.

Comment 1.5: Apart from this, I noticed that "while maximizing" is written twice at the bottom of page 4 and, on page 5, the text refers to figure 1 instead of figure 5.

Response: We thank the Reviewer for catching these typos. The duplicate "while maximizing" has been removed, and the incorrect figure references in the text have been corrected.

Reviewer 2

The authors theoretically propose and experimentally observe the phenomenon of the relativistic Brewster effect in electron-photon scattering. Fundamentally, they demonstrate a vanishing scattering of p-polarized light by relativistic electron beams. These results are novel and interesting, and the theory is preliminarily verified by experiments. Therefore, I believe this

manuscript deserves publication in Nature Communications, provided the following comments and questions are adequately addressed.

Response: We thank the Reviewer for their strong support of the manuscript and the constructive suggestions to improve the depth of the discussion.

Comment 2.1: I suggest that the structure of the electron beam be elaborated upon in greater detail, specifically regarding whether this structure plays a role in the observed effect. The beam structure might influence the orientation of electron-electron interactions within the beam (i.e., whether they are predominantly along the beam axis or perpendicular to it), which could subsequently impact its optical response.

Response: We thank the Referee for the suggestion to discuss more explicitly the effects of electron-beam structure in our experiment. In the revised manuscript, we distinguish between two regimes. In the present experiment, which is in the incoherent scattering regime, the relevant beam structure is the momentum-space distribution of the electrons: transverse emittance, angular divergence, and energy spread. In a coherent or superradiant extension of the interaction, the real-space structure of the electron bunch would also play a central role by determining the collective radiation form factor.

Incoherent Regime (current experiment): The observed relativistic Brewster effect occurs in the incoherent scattering regime. Therefore, the measured optical response is well described as the sum of the intensity patterns emitted by individual electrons. In this limit, the main effect of the beam structure is through the electron momentum distribution, including angular divergence and energy spread, which may themselves be influenced by space-charge forces and other electron–electron interactions upstream of the interaction point. Because the Brewster-like node is a sharp kinematic feature (i.e. it depends on the relative velocities of the electrons and the light), finite angular divergence and energy spread can smear the observed suppression. By Taylor-expanding the differential cross-section near the node, it can be shown that the node is preserved as long as the relative energy spread and angular divergence satisfy $\sigma_\gamma/\gamma \ll 1$ and $\gamma\sigma_\theta \ll 1$, respectively. Both of these conditions are satisfied by the beam parameters in our experiment. We have added a paragraph to the Methods section elaborating on this interplay between beam momentum spread and the experimental observation of the

Coherent Regime: The spatial structure of the beam starts to matter when considering the coherent limits of the interaction, though we emphasize this is not the case in our present experiment.However, the Reviewer's point about the beam structure prompted us to expand the Discussion and examine in greater depth a potentially important extension of the relativistic Brewster geometry.

In conventional head-on inverse Compton scattering, superradiant emission generally requires the bunch length to be comparable to, or shorter than, the emitted wavelength. However, at the relativistic Brewster angle, the Lorentz transformation maps the laser to exactly normal incidence ($\theta_0' = \pi/2$) in the electron rest frame. As a result, the relevant coherence condition involves the transverse structure of the beam suggesting that coherent enhancement may be possible in geometries where a bunch longer than the emitted wavelength still radiates collectively into selected angular modes. In the coherent limit, the bunch form factor would reshape the angular emission pattern and could enhance or suppress specific off-axis components through interference. By tailoring the macroscopic beam geometry, for example through the transverse size, tilt, or crabbing angle of the bunch, one could modify the phase-matching conditions and control the topology of the emitted radiation. This would make the electron bunch a closer dynamical analog to a macroscopic dielectric boundary. We have added a new paragraph to the Discussion describing this mechanism and clarifying that it is a prospective coherent-regime

extension rather than an effect required to explain the present measurements.

Comment 2.2: The authors mention the concept of "moving media." Would it be possible to schematically plot the optical dispersion surface of this electron beam treated as a moving medium? Furthermore, what is the major consequence of the momentum shift in this effect, and how does it evolve with the speed of the electrons?

Response: We utilize the term "moving medium" conceptually to bridge the single-particle Lorentz transformations with the macroscopic language of classical optics (i.e., refractive index and dielectric interfaces). Because the interaction occurs with a free-electron beam rather than a dense plasma, the beam does not possess a complex macroscopic optical dispersion surface in the traditional solid-state sense; i.e. the optical response is governed by the relativistic kinematics set by the electron velocity βc , rather than by a refractive-index dispersion relation. Therefore, a conventional optical dispersion-surface plot would be potentially misleading, as it could imply bulk dielectric or plasma behavior that is not involved in the observed effect.

Regarding the "momentum shift," this can be interpreted in two ways within our framework, both of which we now address in the manuscript:

First, regarding the momentum shift of the scattered light (the relativistic Doppler shift): at the exact relativistic Brewster condition ($\cos\theta_0 = \beta$), the on-axis photon momentum shift simplifies precisely to $p_{\rm out} = p_{\rm in}(1+\beta) \approx 2p_{\rm in}$. As noted in the opening of the Discussion section, the consequence of this specific geometry is that the on-axis Compton edge is strictly limited to frequency doubling of the drive field.

Second, regarding the momentum shift of the electron: as detailed in our response to Comment 2.1, the initial momentum spread of the electron beam (its transverse emittance and energy spread) dictates the smearing of the scattering node. Furthermore, as noted in the Discussion, at much higher incident photon energies, quantum recoil would introduce discrete momentum shifts to the electron, potentially perturbing the exact kinematic balance of the Brewster node.

Comment 2.3: Impedance matching of p-polarized light at the Brewster angle is another common explanation for the Brewster effect. Following this understanding, the Brewster effect has been extensively developed in the field of metamaterials recently. Notable examples include the discovery of the full-polarization omnidirectional Brewster effect (Light Sci. Appl. 13, 262 (2024)) and the anomalous Brewster effect, which enables perfect absorption (Light Sci. Appl. 10, 89 (2021)). The readers would benefit from a comprehensive introduction to these developments and some potential discussion from the perspective of impedance matching.

Response: We thank the Reviewer for highlighting this crucial parallel. The macroscopic impedance-matching perspective provides a highly relevant modern context for our single-particle results. In metamaterials, the Brewster effect is achieved by engineering the electric and magnetic susceptibilities (ϵ, μ) to match the wave impedance of free space, ensuring the radiation from induced electric and magnetic dipoles destructively interferes in the reflection direction.

In our single-particle case, this impedance matching is purely determined by the velocity of the electrons The scattering is governed by the interplay of the electric and magnetic Lorentz forces acting on the moving electron. At the relativistic Brewster angle, the transverse components of the electric and magnetic forces perfectly balance. This kinematic cancellation aligns the net induced dipole exactly along the propagation axis, which suppresses the forward emission. We have added several sentences to the Introduction explicitly citing the suggested papers and drawing this parallel between the generalized impedance matching in engineered metasurfaces

and the electric and magnetic force balancing we observe in the relativistic vacuum interaction.

Comment 2.4: The experimental results shown in Fig. 3 exhibit some discrepancies compared to the theoretical results in Fig. 2. What is the main cause of this deviation? Is it possible to improve the results or add more analyses to bolster confidence in the observation of this phenomenon?

Response: The reviewer may be noting that the measured p-polarized yield in Fig. 3b does not drop to exactly zero unlike the theoretical dip shown in Fig. 2b. However, we clarify that there is no discrepancy between the theoretical predictions (Fig. 2) and the experimental data (Fig. 3).

This distinction arises from the difference between the strictly on-axis theoretical quantity and the finite experimental collection aperture. As we now more explicitly define in the revised caption of Fig. 2, the theoretical "reflectivity" evaluates the scattering efficiency exclusively along the principal scattering axis ($\theta = 0$). Because the fundamental node resides perfectly on-axis, this theoretical reflectivity drops to exactly zero.

In contrast, the experiment (Fig. 3) measures the total photon yield captured by our detector. Because the p-polarized radiation is redistributed into off-axis lobes (as correctly noted by the Reviewer and shown in Fig. 2a), our detector's finite collection aperture ($\gamma\theta_c = 0.5$) inevitably integrates a fraction of this off-axis radiation. The solid curves in Fig. 3 that perfectly overlap with our data points are, in fact, the exact cumulative integrals of the analytic differential cross-sections over this specific aperture. The non-zero minimum is therefore precisely the anticipated behavior predicted by the analytical theory when accounting for the collection angle. We have revised the captions of both Fig. 2 and Fig. 3 to state this distinction between the on-axis reflectivity and the finite-aperture integration more explicitly to prevent any future confusion.

Point-by-Point Response to Reviewers

Reviewer 1

Comment 1: The authors have provided answers to all my comments. Based on their extensive reply, I feel that my initial comments have been reaffirmed in the sense that the direction of the analogy is better understood from microscopic relativistic light-matter interaction to macroscopic polarization optics and not vice versa. As such, calling this the "relativistic Brewster effect" remains problematic in my eyes. A framing such as "analogous to the Brewster effect" would be more honest.

Response: We thank the Reviewer for their careful final reading. We completely agree that precision in the framing is important. In the revised manuscript we have changed the title to "Observation of a relativistic analogue of the Brewster effect in electron–photon scattering", and have gone through the main text, abstract, and conclusion and softened statements of "the relativistic Brewster effect" with "a relativistic analogue to the Brewster effect" (and similar phrasing) to ensure the language reflects the directionality of the physical analogy as the Reviewer suggested.

Comment 2: Similarly, the claim that "this discovery unlocks a new degree of freedom for high-energy photonics" is still overly sensational. I understand the authors try to "sell" their nice and mostly sound results to Nature Communications, but claiming that this result would now allow for people to make structured light at high photon energies using this technique is - in my view - hype. The photon numbers we are talking about here are small and the coherence regime is far away (in particular for high photon energies).

Response: We appreciate this grounding feedback and agree that our original phrasing was overly sensational. To provide a more honest and measured framing, we have removed the phrase "unlocks a new degree of freedom for high-energy photonics".

Comment 3: The additional discussion of a path towards super-radiant emission is a neat idea, but without any simulations backing this up, it is not clear what those conditions would actually be. The yardstick for such kind of claims should be [Malaca et al. Nat. Phot. 18 (2024)], who did extensive simulations to support claims of super-radiance regimes.

Response: We completely agree that rigorous simulations, such as those demonstrated by Malaca *et al.*, will fully validate the discussed superradiant regime. However, providing full multiparticle simulations of this coherent regime is beyond the scope of this experimental observation paper. To ensure the theoretical conditions are clear without overclaiming, we have explicitly referenced Schaap *et al.* (2022). This work contains a general bunching factor formalism that includes the conditions mentioned in this paragraph, albeit implicitly. We have added a sentence noting that a detailed description of this specific "transverse" superradiance phenomenon will be the subject of future work.

Additionally, we thank the Reviewer for pointing us to the reference. We have incorporated a citation to Malaca *et al.*, along with the foundational work on generalized superradiance (Vieira *et al.*), into our revised Discussion. Generalized superradiance from superluminal beam modulation is closely related to our "transverse" superradiance case, as it also allows for coherent emission from bunches longer than the radiation wavelength, which is now briefly mentioned in the revised manuscript.

Reviewer 2

The authors have carefully addressed my comments and revised their manuscript. I now have no more comments.

Response: We thank the Reviewer for their time, constructive feedback, and support of our manuscript during this review process.

Review: Observation of the relativistic Brewster effect in electron–photon scattering

March 17, 2026

This manuscript is clearly written, well structured, and visually effective. The experimental presentation is careful, and the measured polarization dependence and collection-angle dependence appear to be in good agreement with the authors' model. In particular, the data do support the central experimental statement that, at the chosen shallow collision angle, the on-axis signal is strongly suppressed for p-polarized incidence while remaining large for s-polarization, and that the missing on-axis intensity is redistributed into off-axis emission. That said, my main concern is not with the data quality, but with the conceptual framing and novelty claim.

The manuscript presents the result as the "observation of the relativistic Brewster effect" and repeatedly draws an analogy to Brewster reflection at a dielectric interface, where the p-polarized reflected channel vanishes. But strictly, this is not the effect they observe: The authors show that the p-polarized scattering is not eliminated, but instead becomes an annular/lobed distribution with a zero only on axis. Both the main text and supplemental material explicitly state that the cross-section at the special angle takes the form

$$\frac{d\sigma}{d\Omega} = \frac{r_e^2 \sin^2\theta}{\gamma^4(1-\beta\cos\theta)^4}, \qquad \text{see Eq.2 of the main text,}$$

which is zero on axis *but finite off axis*, peaking at a nonzero angle. First, that is not strictly analogous to the Brewster effect. Second, the expression above is derived from the general polarization-dependent relativistic Thomson/Compton cross-section by setting $\phi_0 = \pi/2$ and $\theta_0 = \arccos\beta$. It is thus not a new theoretical result but a particular limit of the standard formalism, corresponding to the familiar p-polarized radiation pattern with an on-axis node and off-axis lobes. The radiation structure is well-documented in the literature. When the authors claim that "this fundamental optical anomaly has never been isolated", they refer to their explicit framing. Many people have looked at the emission and seen the zero, no matter the interaction angle.

What is shown here is a particularly clear and nicely visualized instance of a known polarization-dependent relativistic radiation pattern. I found it highly educational to draw the analogy to Brewster. This makes this a pedagogical achievement to me. While I highly appreciate the analogy, statements like "this

work demonstrates that the foundational principles of polarization optics are not confined to material media but are intrinsic to the relativistic interaction itself" should be revised to avoid misinterpretation. I am sure the authors mean it the right way, but it should be emphasized that it is not that relativistic interaction behaves like polarization optics, but that polarization optics is a special case of (relativistic) light-matter interaction. This is also why I am skeptical of the framing as "relativistic Brewster effect": the analogy is only partial due to the off-axis content.

However, under any reading, their work does not identify a previously unknown property of electron–light scattering. Any standard Compton/Thomson simulation that retains polarization and angular dependence will already produce this node/lobe structure. This is also why I doubt that this result "establish[es] a new framework" or provides "a new degree of freedom for high-energy photonics, enabling the synthesis of exotic vector beams and structured light fields across the electromagnetic spectrum".

Apart from this, I noticed that "while maximizing" is written twice at the bottom of page 4 and, on page 5, the text refers to figure 1 instead of figure 5.
